# Analyses of physiological wrist tremor with increased muscle activity during bench press exercise 

**DOI:** 10.20463/jenb.2019.0001

**Published:** 2019-03-31

**Authors:** Hyewon Son, Jisu Kim, Gyuseog Hong, Wonil Park, Sungjin Yoon, Kiwon Lim, Jonghoon Park

**Affiliations:** 1 Department of Physical Education, Korea University, Seoul Republic of Korea; 2 Physical Activity and Performance Institute, Konkuk University, Seoul Republic of Korea; 3 Convergence Center, LG Electronics, Seoul Republic of Korea; 4 Department of Physical Education, Konkuk University, Seoul Republic of Korea

**Keywords:** tremor, muscle activity, bench-press exercise

## Abstract

**[Purpose]:**

To date, there have been no studies on the response of wrist tremor to increased muscle activity during exercise. This study aimed to evaluate the wrist tremor response with increasing muscle activity during bench press exercise.

**[Methods]:**

Triceps muscle activity and wrist tremor response were measured by electromyography and an accelerometer, respectively, during bench press exercise in 11 healthy men without weight-training experience. Subjects performed bench press at 30% repetition maximum (RM), and the rating of perceived exertion (RPE) and lactate concentration were measured before and after exercise. One week later, an equivalent number of bench presses at 30% RM was performed without weight load as a control trial (CT).

**[Results]:**

RPEs and lactate concentrations significantly increased after resistance exercise (30% RM) from 7.4 to 14.3 and 1.7 to 4.9, respectively (P<.01), but no such difference was observed in the CT. Muscle activity linearly increased during the 30% RM exercise, and wrist tremors were shown to linearly decrease. A strong negative correlation was observed between the two variables (r=−0.88, P<.001).

**[Conclusion]:**

We found that wrist tremors during resistance exercise, as measured using an accelerometer, can be used to predict muscle activity.

## INTRODUCTION

Heart rate is widely used to predict intensity during aerobic exercise^1^. The rating of perceived exertion (RPE), which measures an individual’s subjective intensity, is used to predict intensity during resistance exercise^2^. However, unlike continuously performed aerobic exercises, accurately predicting exercise intensity during intermittently performed anaerobic resistance exercises is difficult and is limited to the use of indirect predictors of intensity. Currently, there are no known indicators that can be used to predict and prescribe intensity during resistance exercise.

Electromyogram (EMG) analysis is commonly used when measuring changes in muscle activity during resistance exercise^3^^,^^4^. Using EMG analysis, information regarding the relationship between EMG signal, force generation, and muscle fatigue can be obtained^3^^,^^4^. Generally, muscle activity displays a tendency to increase when more motor units are incorporated with increasing muscle contraction from increased repetitions with constant intensity. In addition, when muscle activity was analyzed while performing the repetition maximum (RM) during bench press exercise, muscle activity was observed to increase linearly for a relatively long period in relatively low-intensity conditions^5^.

A physiological tremor is an involuntary muscle contraction and relaxation involving oscillations or twitching movements. It is the most common of all involuntary movements and can affect the hand^6^. Tremor has been reported occur due to the effects of motor units, summation of contraction strengths, and the difference between the simultaneous synchronization of motor units and muscle strengths^7^^,^^8^. That is, this type of physiological tremor results from an abnormality in the proprioceptive feedback from the limbs (peripheral nervous system) to the cerebral cortex (central nervous system) while maintaining a limb in a specific position or performing movements against gravity and mechanical forces, even in healthy humans^9^. Kim and Kim^10^ reported that wrist tremor significantly increased when muscle activity decreased due to muscle fatigue after performing an exercise, wherein 2.5-kg weights were repeatedly lifted and lowered using the wrist. The study by Kim and Kim^10^ is the only available research reported to date on the relationship between muscle activity and tremor due to resistance exercise and assessed muscle fatigue at only one timepoint after resistance exercise rather than measuring the tremor during exercise. To date, there have been no reports demonstrating how wrist tremor responds to increasing muscle activity during exercise. In our study, we measured lactate and RPE before and after low-intensity bench press exercise (30% RM) in healthy males and assessed the wrist tremor response using an accelerometer as well as muscle activity during the exercise to determine the correlation between the two variables.

## METHODS

### Subjects

The subjects of this study were 11 healthy men in their twenties without orthopedic illnesses for one year prior, who were not taking medications, and who lacked weight training experience. The purpose and methods of the study were explained to the subjects before the consent form was signed. This research was conducted under the approval from the institutional review board (KUIRB-2018-0097-01). The mean age, weight, height, body fat percentage, and body mass index of the subjects were 25.8±3.0 years, 71.5±9.9 kg, 177.2±5.6 cm, 17.7±4.5%, and 24.9±4.9, respectively.

### Study procedure

One week before the main study was conducted, the weight, height, body composition, and 1 repetition maximum (RM) for bench press were measured. The measurements were taken in the afternoon, and the subjects fasted and did not smoke for 4 h after lunch. At the first measurement (30% RM), the research subjects rested for >20 min by sitting in a chair in the laboratory. After resting, lactate and RPE were measured before exercise. Afterwards, each research participant wore a heart rate monitor and an accelerometer on the wrist, and an EMG electrode was attached to the triceps. Subsequently, heart rate, muscle activity, and wrist tremor were measured while performing the 30% RM bench press. After exercise, lactate and RPE were measured. After 1 week, a control trial (CT) was conducted, where the same number of repetitions performed during the first measurement were performed without weight loads (bar only). All measurements were performed as described for the 30% RM bench press.

### Exercise 

Each research participant was instructed to lie on the bench, spread their feet apart to shoulder-width, touch the floor with the entire bottoms of their feet, and hold the bar a little wider than the width of their shoulder. In the lifting stage of the exercise, the study participant was instructed to lift the bar at an even interval according to the prepared signals. Here, the arms were to be lifted up until completely straight, the back was not to be arched, and the chest was not to be lifted. In the lowering stage, the study participant was instructed to lower the bar at an even interval until the bar almost touched the chest, according to instructions from the assistant. Here, the assistant was instructed to prevent injury of the research participant and provide motivation so that the participant could perform the RM^11^. The wrist tremor amplitude and EMG were measured during one set of all-out bench press exercise.

### Measurements

#### Body composition

Variables, such as weight and body fat percentage, were analyzed using a body composition analyzer based on bioimpedance (Inbody 520, Bio-Space, Korea). Metal jewelry was removed before measurement. Measurement was performed while the fingers, thumb, and bottoms of the subject’s feet contacted the surface of the electrode and arms were spread to an appropriate width.

#### 1RM measurement

1RM was measured using the indirect measurement method^12^. 1RM refers to the muscle strength used against the resistance of the maximum weight that can be lifted at once (i.e., the ability of the muscle). The following equation was used to calculate 1RM:

$$\begin{array}{c} \text{1RM=Wo+}{\text{W}}_{\text{1}}\\ \text{Wo=Weight thought to be a little heavy after sufficient preparatory exercise}\\ \text{W1=Wo*0.025*R }\left(\text{=actual number of repetitions}\right) \end{array}$$

#### Lactate

The concentration of lactate in the blood was measured using a lactate analyzer (Lactate Pro 2, USA). Blood was sampled using the fingertip method.

#### Heart rate

Heart rate was measured using a wireless heart rate monitor (Polar, S610i, Finland). The transmitter (the component that detects heart rate signals and transmits them to the monitor) was placed on the center of the chest of each research participant, and the belt was worn below the chest. The watch showing the heart rate was worn by the assistant to check and record the heart rates. Measurement was performed before, during, and immediately after the exercise.

#### Muscle activity 

EMG measurement was performed using a four-channel wired EMG (Laxtha, Korea). We used surface electrodes and organized the wires connecting the electrodes and the EMG machine in order to prevent motion artifacts, and we also subsequently monitored for any motion artifacts. Electrodes were attached to the triceps to obtain the electrical signals of the muscle for active force. The root mean square (RMS) of muscle activity during exercise was calculated for analysis.

#### Wrist tremor 

Accelerometer data were obtained using three-axis sensors (Accelerometers, Invensense, Korea) attached to the wrist of the dominant arm. The accelerometer was adjusted by calibrating in DC mode before testing in AC mode.

#### Rating of perceived exertion (RPE) 

We explained the Borg 15-point range-proportion scale in detail to the research participants before initiating measurement^13^. The participants were asked to point with their fingers before and immediately after exercise to indicate their rating.

### Statistical analysis

SPSS ver. 21.0 (SPSS Inc., Chicago, IL, USA) was used for all statistical analyses used to test the hypothesis set forth in this study. The mean and standard deviation (SD) were calculated for the variables using descriptive statistical analyses. Paired t-tests were performed to compare and analyze lactate levels and RPE before and after resistance exercise. Tukey’s post-hoc multiple comparisons test was used to compare and analyze the heart rates measured during resistance exercise between subjects by session. Pearson correlations were used to examine the relationship between muscle activity and wrist tremor. Normalized data were used to assess the correlation between muscle activity and wrist tremor. In our study, normalization of ratings was performed to adjust values measured on different scales to a notionally common scale. The significance level for hypothesis verification was set at P<.05.

## RESULTS

**Figure 1:**
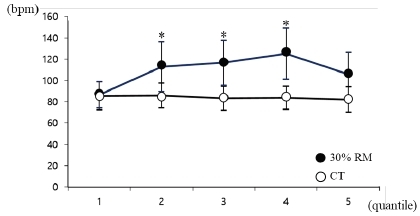
Heart rate during bench press exercise. To evaluate changes in heart rate during resistance exercise, the exercise time was divided into five periods for analysis, as the required exercise time differs between subjects due to differing RMs. * vs. control trial at each session (P<.05). Data are expressed as mean ± SD. RM, repetition maximum.

The RPE of a 30% RM bench press increased significantly after resistance exercise, from 7.4 to 14.3 (P<.01), but did not increase in the CT. At 30% RM, lactate concentrations significantly increased after resistance exercise, from 1.7 to 4.9 mmol/L (P<.01), but did not increase in the CT. To evaluate changes in heart rate during resistance exercise, the exercise time was divided into five periods for analysis, as the required exercise time differs between subjects due to their differing RMs. At 30% RM, a significantly higher heart rate was observed in periods 2, 3, and 4 compared with the CT (P<.05), but no differences were found in the heart rates during exercise in the CT (Figure 1).

**Figure 2:**
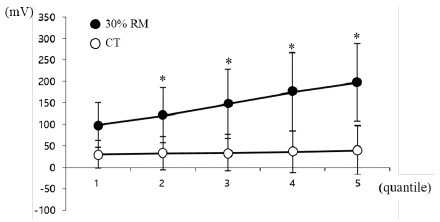
Muscle activity measured by EMG during bench press exercise. To evaluate changes in muscle activity during resistance exercise, the exercise time was divided into five periods for analysis, as the required exercise time differs between subjects due to differing RMs. * vs. control trial at each session (P<.05). Data are expressed as mean ± SD. RM, repetition maximum.

When evaluating changes in muscle activity during resistance exercise, the exercise time was divided into five periods for analysis (Figure 2). Muscle activity increased linearly during the 30% RM exercise, and the values were significantly higher in the 30% RM exercise when compared with the CT for all exercise periods (P<.05).

**Figure 3:**
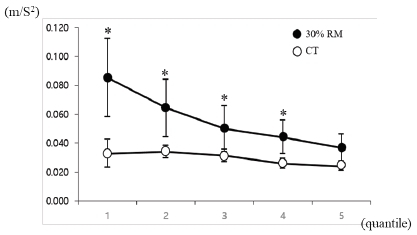
Wrist tremor during bench press exercise measured using an accelerometer. To evaluate changes in wrist tremor during resistance exercise, the exercise time was divided into five periods for analysis, as the required exercise time differs between subjects due to the differing RMs. * vs. control trial at each session (P<.05). Data are expressed as mean ± SD. RM, repetition maximum.

Wrist tremors during exercise linearly decreased in the 30% RM exercise but a difference in response to changes was not observed for the different exercise periods in the CT (Figure 3). The wrist tremor values were significantly higher in periods 1 to 4 in the 30% RM exercise compared to that in the CT (P<.05).

**Figure 4:**
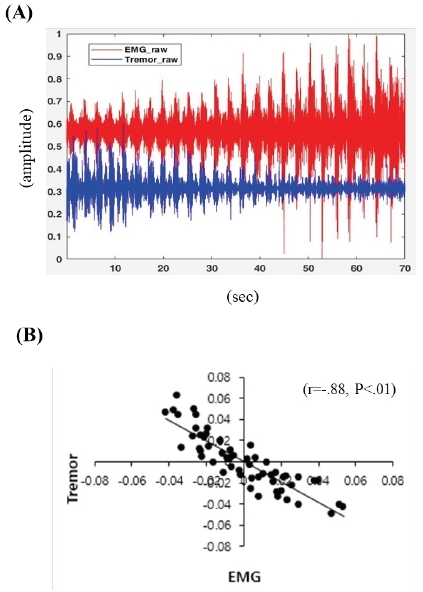
Correlation between muscle activity and wrist tremor during bench press exercise. (A) Waveforms of muscle activity (red) and wrist tremor (blue) of one study participant during the 30% RM exercise. (B) Correlation between muscle activity and wrist tremor during exercise (r=−.88, P<.001). RM, repetition maximum.

Figure 4 shows the waveforms of muscle activity (red) and wrist tremor (blue) of one research participant during 30% RM exercise (Figure 4-A). Analysis of the correlation between muscle activity and wrist tremor during exercise (Figure 4-B) uncovered a strong negative correlation between muscle activity and wrist tremor (r=−.88, P<.001).

## DISCUSSION

Importantly, in our study, we observed that wrist tremor intensity linearly decreased when muscle activity linearly increased during bench press exercises, and these two variables display a strong negative correlation.

Byeon^14^ showed that blood lactate concentrations increased from 2.4 to 4.75 mmol/L when one set of RM leg extensions was performed at 30% RM. In our study, subjects performed one set of bench presses at RM at a constant intensity, and lactate concentrations increased from 1.7 to 4.9 mmol/L. Although the two studies used resistance exercises of equal intensity with different muscles, the observed lactate values were similar. In our study, RPE was found to be 14.3 on a scale of 6 to 20, demonstrating that the subjective intensity felt by the research participants was similar to that of other studies^15^. In our study, changes in heart rate significantly increased during exercise at 30% RM. Byeon^14^ observed heart rates of 71.9 and 92.7 bpm before and after performing one set of exercises, respectively. In our study, heart rate tended to increase from 87 to 125 bpm at the initial measurement and decrease to 106 bpm near the end of exercise. The results of our study uncovered a similar pattern in the increase in heart rate during RM performance during low-intensity resistance exercise^16^^,^^17^. In our study, we performed a low-intensity RM that gradually increased muscle activity over a relatively long time period to observe the relationship between muscle activity and wrist tremor during exercise. Our results verify that our study was sufficiently sensitive to the effects of resistance exercise.

In our study, muscle activity linearly increased until termination of the exercise without suppression of muscle activity due to muscle fatigue with an increase in repetitions at 30% RM. The consistent increase in muscle activity during resistance exercise in our study is considered to be the potential explanation for the large observed EMG, due to the force exhibited by the continuous increase in muscle activity. Our study also revealed a decline in wrist tremor with increasing muscle activity during bench press exercise, and a strong negative correlation was observed between these two values. Hand tremor is influenced by muscle elasticity, muscle tension, and neural feedback; among those, neural feedback plays a definitive role in the presence of hand tremors^18^. Through this mechanism, a 8–12 Hz vibration of the hand was reported to be a mechanistic tremor due to the mass load of the hand and was related to the muscle activity of the extensor muscle^18^. Novak and Newell^19^ also reported that the physiological tremor occurring within 8–12 Hz was due to variability in isometric force control. Similarly, our research also found high values for wrist tremor during exercise, though not for isometric control, in the low frequency range of 7–13 Hz (data not shown). It is inferred that the changes in wrist tremor during exercise observed in our study are the result of neural feedback activation due to an external load.

Some previous findings on tremor have been reported in relation to muscle fatigue^10^^,^^20^. In a study by Li et al^20^, healthy subjects performed a sustained maximal grip contraction test to evaluate muscle fatigue and tremor. Data were analyzed in five consecutive 5-s periods to identify changes in fatigue and tremor over time. The maximal exercise induced an increase in physiological tremors during short periods. Conversely, in our study, the wrist tremor amplitude decreased during one set of all-out bench press exercise, even though muscle activity increased. Plausible explanations for the different results regarding tremor changes during exercise between the Li et al. study^20^ and our study may include: 1) different types and duration of exercise between the two studies. The main arm muscle groups used in the exercises differed between Li et al. (grip contraction)^20^ and our study (bench press). In addition, Li et al.^20^ utilized a maximal isometric exercise, while we utilized an all-out isotonic bench press exercise. The duration of the trial was around 10 seconds in the Li et al. study^20^, while our trials were longer (approximately 60-80 seconds). 2) In our study, the wrist tremor amplitude was relatively higher in the first and second quantiles compared with the other quantiles. It may be that prior movement in the earlier phase of the bench press exercise results in a transient reduction in frequency and an increase in amplitude of the physiological tremor by transiently reducing joint stiffness, despite unchanged force^21^. The movement in the later phase may have optimized the amplitude of the physiological tremor against the external weight with increased joint stiffness. Allum et al.^22^ suggested that an increase in motor unit recruitment resulted in tremor in unfatigued muscles. Ebenbichler et al.^23^ also found that the force power spectra across frequencies (6±20 Hz) at the beginning of the fatiguing contractions was greater when compared to those at the end when performing a 30% maximum voluntary contraction. 3) In our study, in the earlier phase of bench press exercise, arm muscles may have been less adapted and thereby resulting in a transient abnormality in the proprioceptive feedback from the limbs to the cerebral cortex while trying to maintain a limb at a specific position or movement. Our study demonstrated a strong negative correlation between muscle activity and wrist tremor during one set of all-out bench press exercise with low intensity. To the best of our knowledge, we are the first to report changes in wrist tremor occurring with an increase in muscle activity during resistance exercise, and thus demonstrating that wrist tremor can be used as an index to predict muscle activity during resistance exercise.

The results of this study showed that measurement of wrist tremor during resistance exercise using an accelerometer can be used as an index to predict muscle activity. In future research, wrist tremor should be measured according to the intensity of the resistance exercise, and the wrist tremor response should be comprehensively examined in various types of resistance exercises and situations in which muscle fatigue is induced.

## References

[1] Achten J, Jeukendrup AE. (2003). Heart rate monitoring: applications and limitations. *Sports Med*.

[2] Hollander DB, Durand RJ, Trynicki JL, Larock D, Castracane VD, Hebert EP, Kraemer RR. (2003). RPE, pain, and physiological adjustment to concentric and eccentric contractions. *Med Sci Sports Exerc*.

[3] Sale DG. (1987). Influence of exercise and training on motor unit activation. *Exerc Sport Sci Rev*.

[4] Kraemer WJ, Fleck SJ, Evans WJ. (1996). Strength and power training: physiological mechanisms of adaptation. *Exerc Sport Sci Rev*.

[5] Yoo K, Ko S. (2012). Optimal Repetition for Muscle Power, iEMG, MPF, and Total Work during Bench Press with Exercise Intensities. *The Asian Journal of Kinesiology*.

[6] Lakie M, Vernooij CA, Osborne TM, Reynolds RF. (2012). The resonant component of human physiological hand tremor is altered by slow voluntary movements. *J Physiol*.

[7] Lakie M, Vernooij CA, Osler CJ, Stevenson AT, Scott JP, Reynolds RF. (2015). Increased gravitational force reveals the mechanical, resonant nature of physiological tremor. *J Physiol*.

[8] Marshall J, Walsh EG. (1956). Physiological tremor. *J Neurol Neurosurg Psychiatry*.

[9] Flament D, Vilis T, Hore J. (1984). Dependence of cerebellar tremor on proprioceptive but not visual feedback. *Exp Neurol*.

[10] Kim Y, Kim J. (2014). Analysis of Muscle Fatigue-Induced Tremor on Wrist Motion. *International Journal of Precision Engineering and Manufacturing*.

[11] Earle RW. Weight training exercise prescription. In Essentials of personal training symposium workbook.

[12] O’Conno B, Simmons J, O’Shea P. (1989). Weight Training Today.

[13] Robertson RJ. (1982). Central signals of perceived exertion during dynamic exercise. *Med Sci Sports Exerc*.

[14] Byun J. (2018). Effects of Acute Traditional Resistance Exercise with KAATSU on Heart Rate, Blood Pressure, RPE and Blood Lactate. *Korea Sports Association*.

[15] Lagally KM, Robertson RJ, Gallagher KI, Goss FL, Jakicic JM, Lephart SM, McCaw ST, Goodpaster B. (2002). Perceived exertion, electromyography, and blood lactate during acute bouts of resistance exercise. *Med Sci Sports Exerc*.

[16] Fahs CA, Rossow LM, Seo DI, Loenneke JP, Sherk VD, Kim E, Bemben DA, Bemben MG. (2011). Effect of different types of resistance exercise on arterial compliance and calf blood flow. *Eur J Appl Physiol*.

[17] Brandner CR, Kidgell DJ, Warmington SA. (2015). Unilateral bicep curl hemodynamics: Low-pressure continuous vs high-pressure intermittent blood flow restriction. *Scand J Med Sci Sports*.

[18] Bhidayasir R. (2005). Differential diagnosis of common tremor syndromes. *Postgrad Med J*.

[19] Novak T, Newell KM. (2017). Physiological tremor (8-12Hz component) in isometric force control. *Neurosci Lett*.

[20] Li K, Hogrel JY, Duchêne J, Hewson DJ. (2012). Analysis of fatigue and tremor during sustained maximal grip contractions using Hilbert-Huang Transformation. *Med Eng Phys*.

[21] Reynolds R, Lakie M. (2010). Postmovement changes in the frequency and amplitude of physiological tremor despite unchanged neural output. *J Neurophysiol*.

[22] Allum JH, Dietz V, Freund H-J. (1978). Neuronal mechanisms underlying physiological tremor. *J Neurophysiol*.

[23] Gerold R. (2000). Ebenbichlera, Josef Kollmitzera, Zeynep Erimb, Wolfgang N. Lo Èscherc, Katharina Kerschana, Martin Poschd, Thomas Nowotnya, Andreas Kranzla, Christian Wo Èbere, Thomas Bochdanskyf. Load-dependence of fatigue related changes in tremor around 10 Hz. *Clinical Neurophysiology*.

